# Can Bottom Sediments Be a Prospective Fertilizing Material? A Chemical Composition Analysis for Potential Reuse in Agriculture

**DOI:** 10.3390/ma14247685

**Published:** 2021-12-13

**Authors:** Karolina Matej-Łukowicz, Ewa Wojciechowska, Joanna Strycharz, Marta Szubska, Karol Kuliński, Jacek Bełdowski, Aleksandra Winogradow

**Affiliations:** 1 Faculty of Civil and Environmental Engineering, Gdańsk University of Technology, Narutowicza 11/12, 80-233 Gdańsk, Poland; ewa.wojciechowska@pg.edu.pl (E.W.); joannastrycharz1997@gmail.com (J.S.); 2 Institute of Oceanology of the Polish Academy of Sciences, Powstańców Warszawy 55, 81-712 Sopot, Poland; szubi@iopan.pl (M.S.); kroll@iopan.pl (K.K.); hyron@iopan.pl (J.B.); olcia@iopan.gda.pl (A.W.)

**Keywords:** retention tanks, nutrient recovery, C/N ratio, Fe/P ratio, circular economy, sustainable agriculture

## Abstract

Every year, huge amounts of bottom sediments are extracted worldwide, which need to be disposed. The recycling of bottom sediments for soil fertilization is in line with the long-promoted circular economy policy and enables the use of micro and macronutrients accumulated in sediments for soil fertilization. When considering potential agricultural reuse of the dredge sediments, the first necessary step should be to analyze whether the heavy metal content meets the obligatory criteria. Then, the contents of valuable elements required for plant growth and their ratios should be assessed. In this study, the content of nitrogen, organic carbon, phosphorus, and potassium was tested and iron, sulfur, calcium, and magnesium were also analyzed along vertical profiles of sediments extracted from four urban retention tanks in Gdańsk (Poland). The sediments were indicated to have a low content of nutrients (Ntot 0.01–0.52%, Corg 0.1–8.4%, P_2_O_5_ 0.00–0.65%, K 0.0–1.0%), while being quite rich in Fe and S (0.2–3.3%, 0.0–2.5%, respectively). The C/N ratio changed in the range of 17.4–28.4, which proved good nitrogen availability for plants. The mean values of the Fe/P ratio were above 2.0, which confirms that phosphorus in the sediments would be available to the plants in the form of iron phosphate. To summarize, the bottom sediments from municipal retention reservoirs are not a perfect material for soil fertilization, but they are a free waste material which, when enriched with little cost, can be a good fertilizer. Future research should focus on cultivation experiments with the use of sediments enriched with N, P, Corg.

## 1. Introduction

Natural fertilizers have been applied since ancient times as a basic material to enrich soil nutrient content [1]. Nowadays, they have been mostly replaced by mineral fertilizers produced from fossil or synthetic elements. The biggest problem with the availability of a fertilizer ingredient relates to phosphorus. The main source of phosphorus in these fertilizers are phosphate rocks, whose resources are predicted to be exhausted by the end of the 21st century if their rate of extraction does not decrease [2]. Already in 2014 the European Commission officially recognized this non-renewable raw material as critical [3]. It was also pointed out that mineral fertilizers containing phosphorus derived from phosphorus rocks are enriched with naturally occurring cadmium. At the same time, it was also predicted that the decrease in production of mineral fertilizers will restrict toxic emissions to the environment, lower energy consumption, and reduce waste generation in the energy production process. The use of alternative materials as fertilizers in agriculture corresponds to Sustainable Development Goals (2.4, 12.2).

Sewage sludge, industry waste, and wastewater are all considered to be sources of element recovery (mainly phosphorus). To date, most of the research work has focused on the utilization of sewage sludge from municipal wastewater treatment plants [4,5] or sediments derived from fish breeding [6,7]. For instance, Haque et al. (2016) [5] conducted studies using fish pond sediment as a potential fertilizer and demonstrated its potential ability to improve grass growth. In this context, the sediments that are deposited in urban retention tanks constructed for flood protection or recreation reasons as well as in rivers [8,9] could also be considered as material containing elements that may be useful for plant cultivation. According to the reports, 200 million m^3^ of bottom sediments are mined annually in Europe [10]. The build-up of bottom sediments needs to be regularly removed to maintain the storage and retention capacity of retention tanks [11], which creates a considerable problem in terms of how to use the periodically dredged materials [12]. On the other hand, the bottom sediments could be viewed as a potential source of microelements and nutrients. The possibility of reusing sediments deposited in urban retention tanks as fertilizers would hit two environmental problems at the same time. In addition, it would contribute to closing the material and resource cycle and to decreasing the consumption of artificial fertilizers. However, those double benefits could be achieved only if a comprehensive analysis of bottom sediments composition reveals their potential utility as fertilizers.

The composition of bottom sediments deposited in urban retention reservoirs is directly affected by the quality of the stormwater runoff, which contains pollutants washed off from the catchment area. Investigations of the composition of stormwater in cities were carried out in numerous studies [13,14,15,16]. Plant cultivation and fertilization is considered to be the major source of phosphorus and nitrogen [17]. In the urban catchment area, urban horticulture, lawn, and plants fertilization in parks and gardens are nutrient sources in the stormwater runoff [18]. Other sources of phosphorus and nitrogen in stormwater runoff are animal manure, atmospheric deposition [19], car detergents, and organic wastes, such as grass clippings and leaves [20]. The combustion of fossil fuels is considered to be the main source of sulfur, which can enrich the rainfall (acid rains) or form atmospheric deposition during dry periods, which is subsequently washed off by runoff [21]. Salts used in road de-icing can also be a source of iron, potassium, sulfur, calcium, and magnesium. Iron can also come from wheel balance weights, and sulphates from concrete surfaces [22]. The aim is to introduce ecological fuels or electric cars [23,24,25].

Recently, Renella (2021) [26] made a general analysis of whether the dredged sediments are legally usable and used, putting emphasis on the great potential of these sediments in agriculture and the circular economy. Kiani et al. (2021) [27] also demonstrated the positive effect of sediment from the eutrophicated Mustijärv lake, which is located in a forest area, on ryegrass growth.

However, studies of bottom sediments from urban storage reservoirs have not been conducted in terms of their use as a fertilizer. Therefore, the aim of this paper is to examine the bottom sediments from retention tanks in an urban area, as a potential low-cost source of nutrients and microelements to support plant growth. This aspect, to our knowledge, was unaddressed in former research, while the previously mentioned studies indicate that it may be justified.

The basic constituents of fertilizer are nitrogen, phosphorus, potassium, and organic matter, which are essential for plant growth. On the other hand, the elemental contents of carbon, sulfur, iron, and calcium are also important since they determine the uptake of nutrients, proper development of the plants [28], and/or protection against parasites [29]. Therefore, the contents of nitrogen, phosphorus, potassium, and sulfur, as major mineral constituents of prospective fertilizer, as well as carbon, calcium, and magnesium as elements determining the soil pH, should be examined in bottom sediments. Additionally, the analysis of bottom sediment composition should be performed in view of the Council Directive Protection of the Environment, and in particular of the soil, when sewage sludge is used in agriculture, although this mostly refers to municipal sewage sludge. However, it can also be applied to other types of sediments and provides important restrictions, for instance, the permissible contents of heavy metals [30].

Nitrogen is one of the basic nutrients indispensable for plant growth and is highly mobile in soil and in plant tissues [31]. Optimal growth and development of plants requires a proper C/N ratio [32]. When the nitrogen content is high and the C/N ratio ranges from 1 to 20, mineralization processes prevail. Otherwise, if the C/N ratio exceeds 20, the rate of organic matter decomposition decreases and immobilization becomes a major process. Brust (2019) reports that a C/N ratio exceeding 35 inhibits the activity of soil microbes, which results in stopping both the mineralization and immobilization of organic matter [33].

Fertilizing materials should also be rich in phosphorus. The stability of phosphorus deposition in sediments depends on the type of chemical bonding [34]. According to Kahiluoto et al. (2015), an abundance of this element in sediments is determined by their oxidation as well as iron, sulfur, and manganese content [35]. Martynova (2011) proved that when the Fe/P ratio is below 2, the phosphates remain dissolved in water, but when the ratio increases, they precipitate together with iron and become phytoavailable for plants [36].

Potassium deficiencies are depicted as one of the most relevant causes (along with acidification) of low soil fertility and they also limit nitrogen transformation to proteins. The content of this element in soil is linked to the content of calcium and magnesium. Potassium in fertilizers is commonly present in the form of potassium chloride, which is a product of processed potassium salts.

Sulfur is recognized as another relevant nutrient for plants. Kurmanbayeva et al. (2021) confirmed that sulfur abundance in fertilizers ameliorates ripening and cereal plant productivity [37]. The role of sulfur in slowing down oxidation processes in plants while boosting reduction processes has been demonstrated [38]. Sulfur interacts with phosphorus as well as nitrogen and it is an important component of fertilizing materials since its presence influences plants’ nitrogen uptake. Moreover, sulfur deficiency compromises plants’ resistance to pathogens [39]. Mineral multi-component fertilizers available on the European market sulfur constitute from 3 to 24%. On the other hand, shortages of sulfur fertilizers are reported worldwide [40].

Bottom sediments, as a by-product of the operation of flood protection systems, are regularly extracted and need to be disposed. A comprehensive analysis verifying the composition of bottom sediments from urban waterbodies in terms of their potential as a fertilization material have not yet been performed. Therefore, the objective of our study was to analyze the contents of organic carbon nutrients (N, P, and K) and other relevant elements (S, Fe, Mg, and Ca) as well as elemental ratios (C/N, Fe/P) in sediments deposited in urban retention tanks to elucidate the possibility of their utilization to increase crop productivity in agriculture. Bottom sediments from four retention tanks in an urban area, from eight sampling points (two sampling points at each tank) were examined. Altogether, 93 bottom sediment samples (sediment vertical profiles) were collected and later divided into subsamples corresponding to deposited layers.

The conducted research aims to answer the following questions, which have not been addressed by the scientific literature to date: 1. Which elements, and how much of them, are present in the sediments from urban retention reservoirs? 2. Are bottom sediments from municipal retention ponds a significant source of phosphorus, as a particularly desirable fertilizer component? 3. Are nitrogen and phosphorus in sediments present in a form which is available for plants? 4. Does the elemental composition of bottom sediments qualify them to be used as a fertilizer? 5. What are the legal aspects of sediment reuse in agriculture?

These studies constitute the first part of the research—i.e., identification of the possibility of reusing bottom sediments for plant fertilization on the basis of elemental composition analysis. Future research work will focus on plant cultivation experiments using fertilizing material based on the analyzed sediments.

## 2. Materials and Methods

### 2.1. Characteristics of Retention Tanks

Samples of bottom sediments were collected from four retention reservoirs located in the city of Gdańsk. There are as many as 23 streams with a total length of 78.03 km in the city and as many as 50 retention reservoirs with a total volume of about 130,000 m^3^.

**Figure 1:**
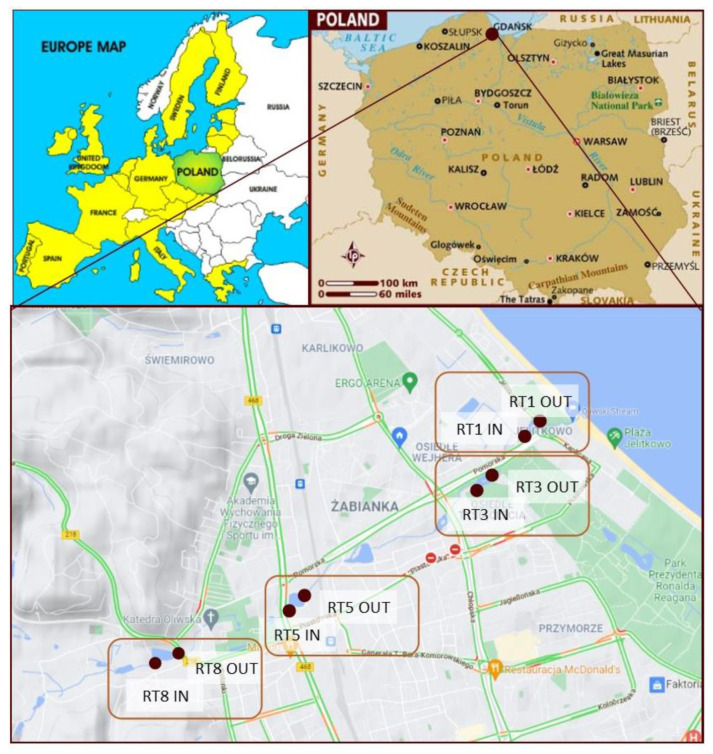
Location of the retention tanks and sampling points.

The samples were collected from tanks in the Oliwski Stream. It is the longest watercourse within the city (9.57 km), with a catchment area of 28.7 km^2^. It acts as a receiver of rainwater from the city and outflows directly to the Baltic Sea. Along the length of the Oliwski Stream, there are 13 retention tanks that protect the city against floods and play a recreational role. Four retention reservoirs were selected for the analysis, marked in Figure 1. The choice was dictated by the location of the reservoirs in relation to residential buildings and roads with the highest traffic, as potentially the most polluted. The characteristics of the tanks are presented in Table 1. The names of the reservoirs refer to the names of the streets on which they are located. Retention reservoirs are emptied on average every 5 years, and the tests were performed 4 years after dredging.

### 2.2. Sampling Method

The samples were collected in the summer of 2017 from four retention tanks along the Oliwski Stream. Samples were collected in the inflow and outflow of the tanks to verify possible variability. The numbers of the collection points are presented in Figure 1. The samples were collected using a sampler made for this purpose, which was hammered into the bottom of the tank to collect sediments. Then, after closing the valve, the sampler was removed from the reservoir and placed on a stand. Thereafter, the core was divided into samples—layers 5 cm thick. The number of samples depended on the height of the core, and therefore the amount of sediment deposited in the tank. A total of 93 samples were taken for the analysis.

### 2.3. Measurement Methodology

The content of phosphorus, iron, potassium, sulfur, magnesium, and calcium in the sediments was determined using a “non-destructive” method that relies on X-ray fluorescence spectroscopy (XRF). The method is widely used to analyze the chemical composition of sediment cores [41,42]. The fluorescence spectrometer S1 Titan 600 from Bruker Poland was used for the research. The samples, previously dried and homogenized, were placed in special plastic containers, lined with a disposable film to ensure the transmission of X-rays to the sample. After calibration with a standard sample, the containers with sediments were placed in the radiation-impermeable element of the measuring apparatus. As they pass through the sample, the rays from the X-ray tube cause two types of reaction: Absorption or scattering. The result was a photoelectric effect and fluorescence radiation, which was individual for each sample and a source of information regarding its composition. All the tests described below were performed in triplicate on each sample. The total nitrogen content was measured on a Flash EA 1112 Series elementary analyzer coupled with a Delta V Advantage IRMS mass spectrometer. The total nitrogen and organic carbon content was measured in an Elemental Analyzer Flash EA 1112 Series combined with the Isotopic Ratio Mass Spectrometer IRMS Delta V Advantage (Thermo Electron Corp., Karlsruhe, Germany). Combustion procces temperatures were as follows: Oxidation at 1020 °C, followed by reduction over copper at 680 °C.

### 2.4. Statistical Analyses

Statistical analyses including the determination of minimum, maximum, mean, median, and standard deviation values were performed using the Statistica 13.1 program. The program also calculated the Pearson correlation coefficients and performed the remaining statistical analyses.

## 3. Results

### 3.1. Geochemical Background

Environmental contamination happens when the concentration of a given chemical substance is above the background. The geochemical background (also referred to as natural background) in environmental sciences is defined as a concentration of a given substance that would be present in the environment without anthropogenic impact [43]. Lis and Pasieczna (1999) determined the values of geochemical background for 22 Elements, including the five analyzed in the bottom sediments of the Oliwski Stream tanks (Table 2).

**Figure 2:**
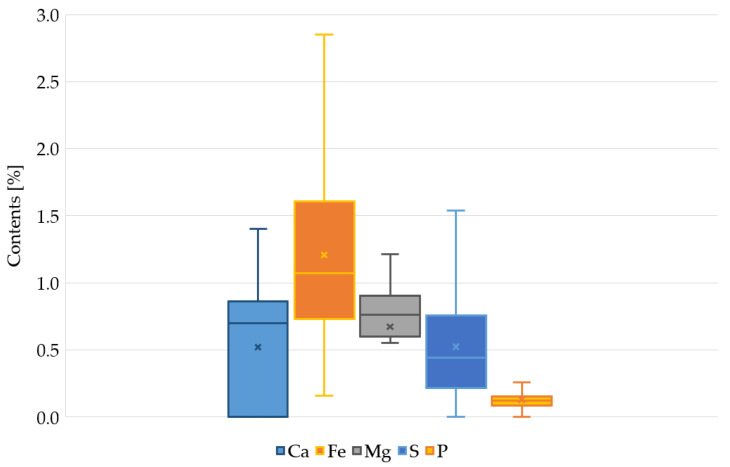
Range of min-max variability of Ca, Fe, Mg, P, and S in bottom sediments of the Oliwski Stream tanks.

Figure 2 shows the range of changes in the content of elements. Taking into account the range of calcium changes, in 62% of the samples the content of this element was higher than the geochemical background for the local soils. In the remaining samples, no calcium content was detected. In a similar analysis for Fe and P, in 84% of the samples the content was higher than the background. The content of Mg and S in almost all samples indicated that the sediments were enriched with this element, compared to the local soils.

### 3.2. Nitrogen and Organic Carbon Content

**Figure 3:**
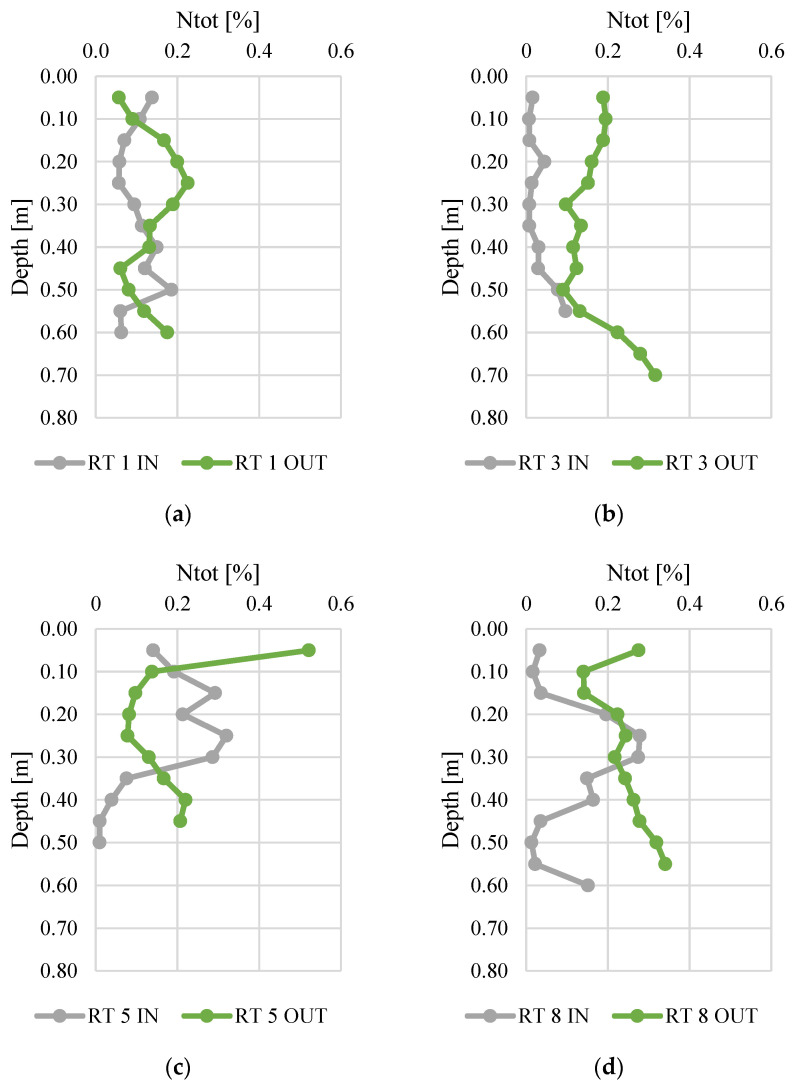
Total nitrogen content along vertical profiles of bottom sediments collected from urban retention tanks. (**a**) RT 1; (**b**)RT 3;(**c**) RT 5;(**d**) RT 8.

**Figure 4:**
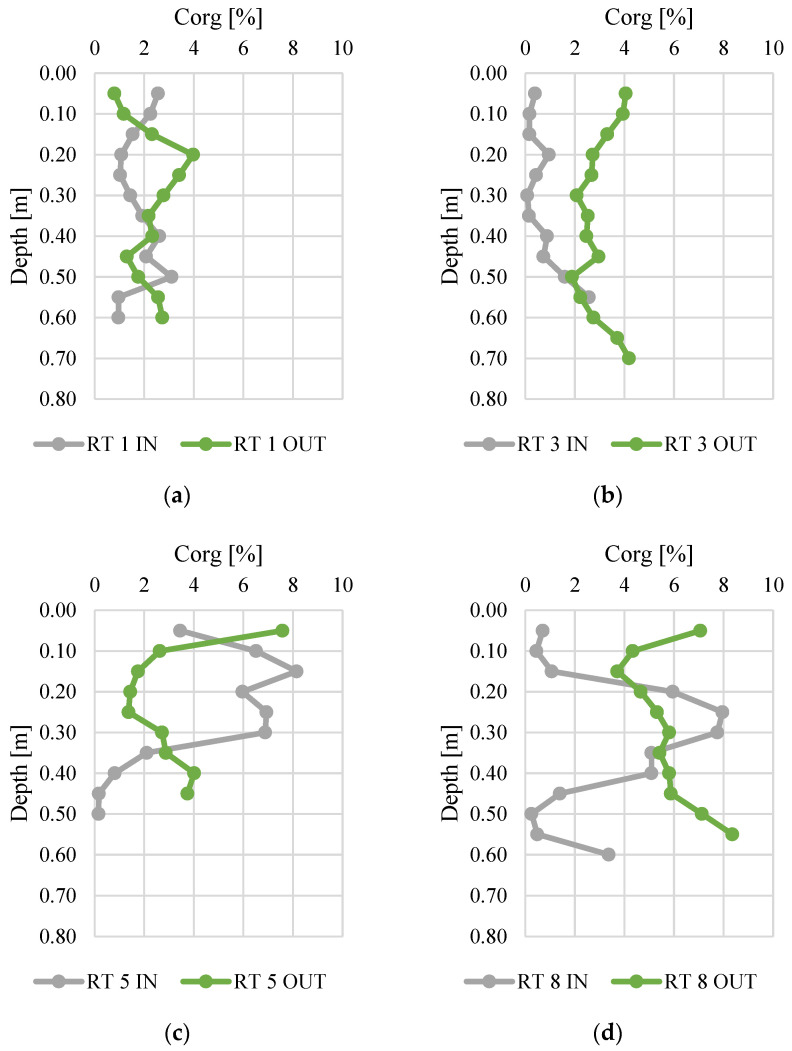
Organic carbon content along vertical profiles of bottom sediments collected from urban retention tanks. (**a**) RT 1; (**b**)RT 3;(**c**) RT 5;(**d**) RT 8.

Figure 3 and Figure 4 present changes in total nitrogen and organic carbon content along the vertical profiles of bottom sediments. The contents of organic carbon varied from 0.01 to 0.52%. The highest average content was found at RT 5 OUT (mean 0.15%, median 0.14%), while the sediments collected at RT 3 IN had the lowest N content (average 0.03%, median 0.01%). The maximum content (0.52%) was (average 5.57%) similar to manure or slurry, confirmed by the values of fertilizer equivalents of nitrogen [45,46]. The organic carbon content varied from 0.1 to 8.4%, and again the highest content was detected in samples from RT8, which was located in the forested, non-urbanized part of the Oliwski Stream catchment. Sediments from RT3 had the lowest organic carbon content (mean 0.7%, median 0.4%). In RT3 and RT8, the Ntot content was definitely higher at OUT. However, in order to verify the reasons for this, it would be necessary to extend the research to an analysis of redox potential, temperature, and pH. Likewise, in most collected sediment profiles, the content of organic C was higher at OUT.

### 3.3. Contents of Phosphorus in Relation to Iron and Sulfur

Following the previously described elemental relationships and ratios, in this paragraph, the phosphorus concentration in bottom sediments along with iron and sulfur was presented.

**Figure 5:**
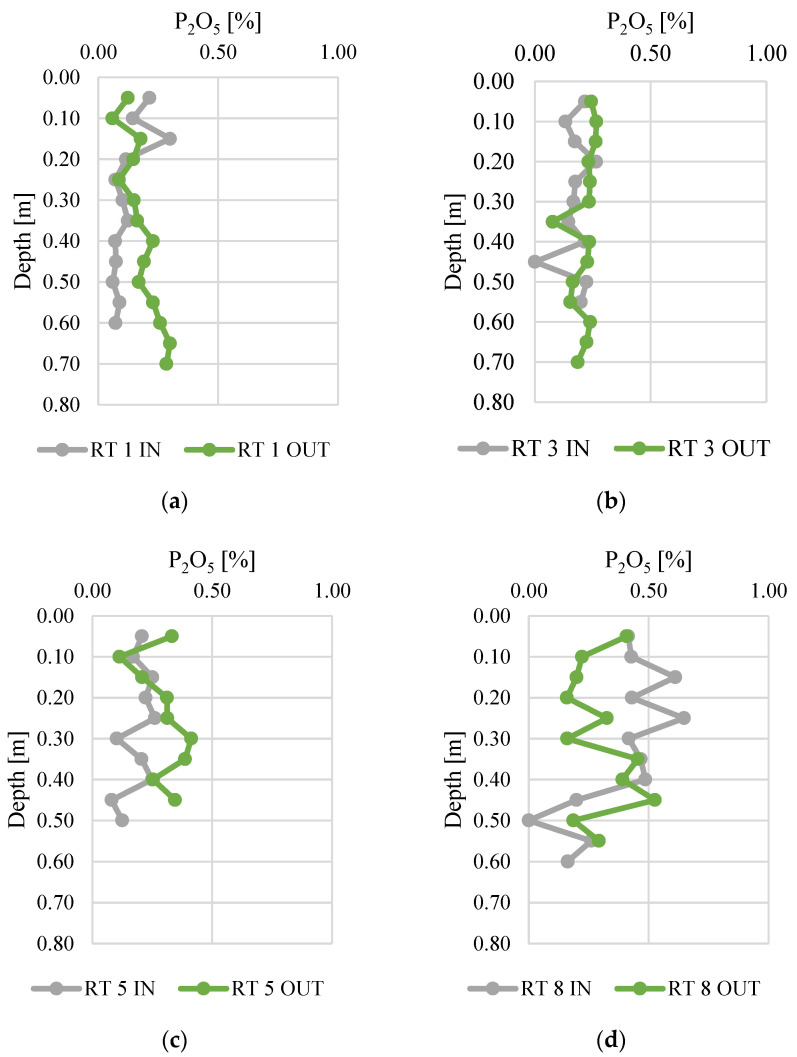
Phosphorus content along vertical profiles in bottom sediments collected from urban retention tanks. (**a**) RT 1; (**b**)RT 3;(**c**) RT 5;(**d**) RT 8.

Phosphorus concentrations in the vertical profiles of bottom sediments are presented in Figure 5. The results are presented in the form of P_2_O_5_, which is also used to express the P content in fertilizers. The contents of P_2_O_5_ ranged from 0.0 to 0.6%, which corresponds to 0.0–0.36% of phosphorus. The highest concentrations of phosphorus were present in RT8, especially at the sampling point located near the inflow (the difference between IN and OUT was 0.08%). Otherwise, in RT1, RT3, and RT5, higher contents of phosphorus were observed near the outflow (the differences between IN and OUT were 0.05, 0.04 and 0.11%, respectively). The lower P contents at the outflow could be due to retention and longer contact time, coupled with higher temperatures (sampling was conducted in summer). These conditions favor the release of P compounds from sediments to the water column (Bartoszek 2007). The lowest standard deviation of the phosphorus concentration measurement results was found at RT 3 OUT (0.029), while the highest standard deviation was at RT 8 IN (0.106). This probably indicates fluctuations in the inflow of phosphorus to the reservoirs. The highest fluctuations of P_2_O_5_ were detected in reservoir no. 8, which is located at the edge of the forested area and urban catchment. Moreover, it is directly below the city zoo, which is also partly located in the forest. Therefore, the catchment of this retention tank differs significantly from the others and can be more prone to some seasonal fluctuations of phosphorus inflow.

**Figure 6:**
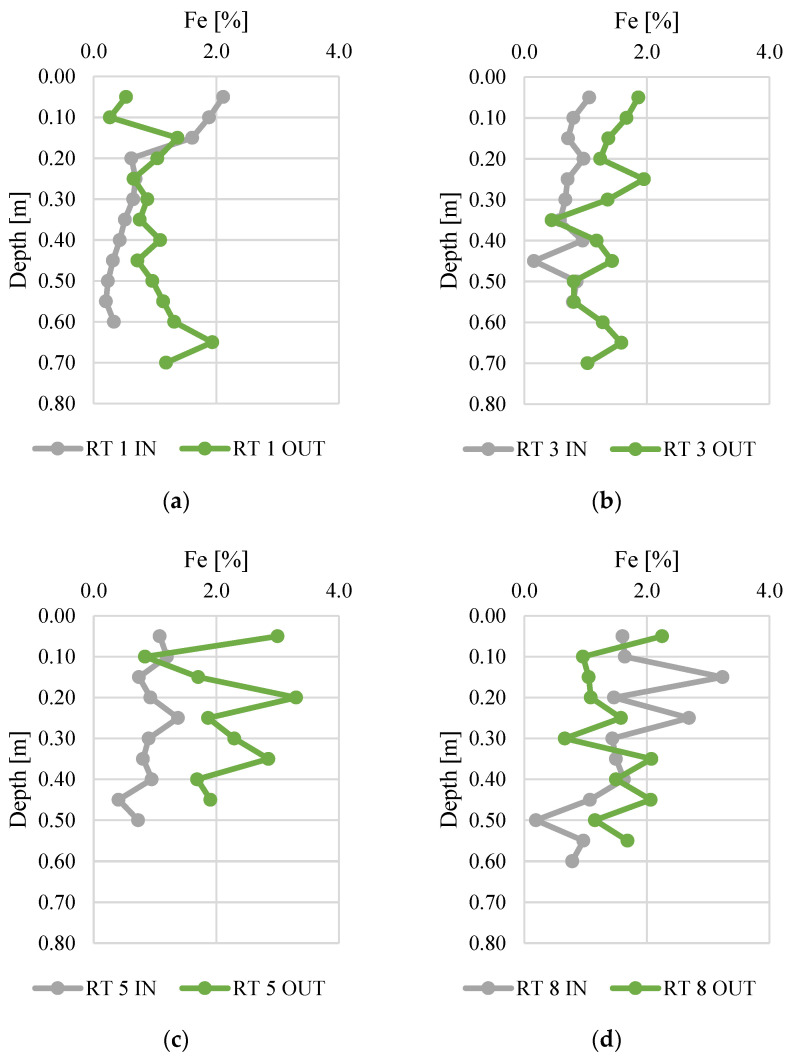
Iron content along vertical profiles in bottom sediments collected from urban retention tanks. (**a**) RT 1; (**b**) RT 3; (**c**) RT 5; (**d**) RT 8.

**Figure 7:**
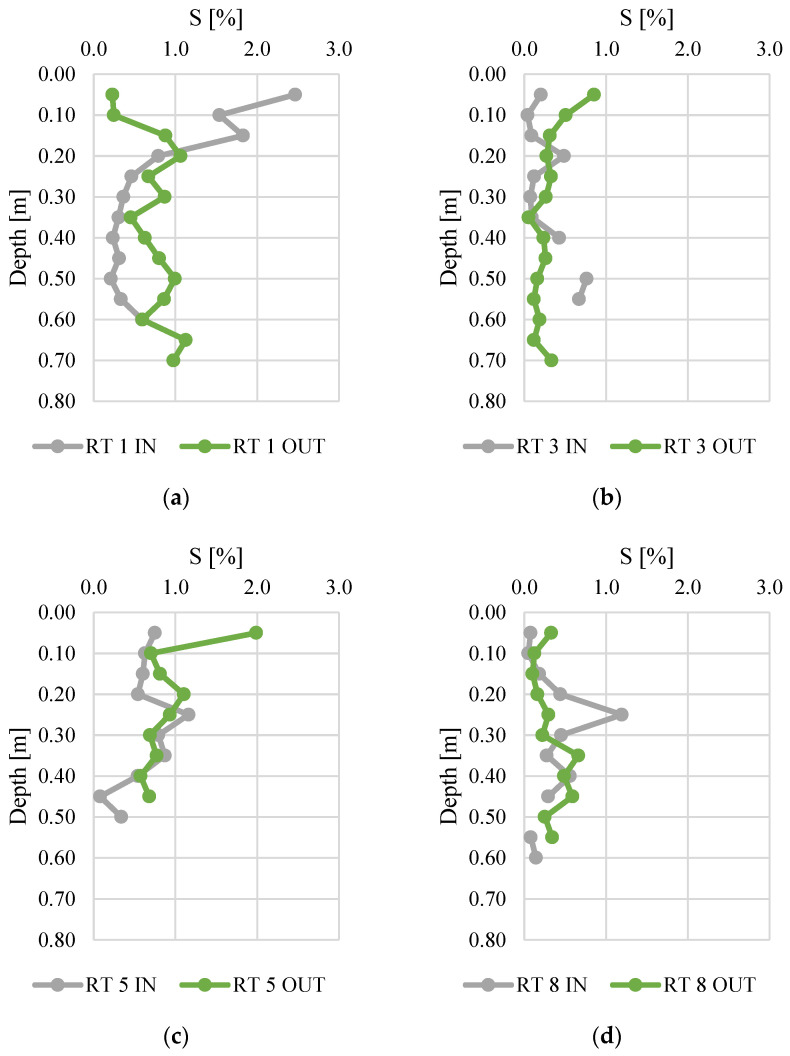
Sulfur content along vertical profiles of bottom sediments collected from urban retention tanks. (**a**) RT 1; (**b**) RT 3; (**c**) RT 5; (**d**) RT 8.

The bioavailability of phosphorus deposited in sediments was analyzed in relation to the content of iron (Figure 6) and sulfur (Figure 7). The Fe content ranged from 0.2 to 3.3%, and was the highest in bottom sediments sampled at RT 8 OUT. At this sampling point, the average concentration in the vertical profile was 2.2%. The samples collected from RT 1 IN and RT 3 IN had the lowest Fe content (average content in the vertical profile was 0.8% for both sampling sites). There was a general trend for higher Fe content at the OUT points. The value of the standard deviation of iron concentration shows a similar, slight variation of five points (mean standard deviation 0.5), but this was larger at RT 5 OUT, RT 8 IN, and RT 8 OUT (1.13, 0.88, 0.71, respectively).

The sulfur content ranged from 0.0 to 2.5%. The bottom sediments collected at RT 1 IN had the highest S content (average for the vertical profile was equal to 0.8%). High average and median values of S content were also calculated for RT 5 OUT. There were no typical fluctuations of S content in vertical profiles, since usually the S content dropped significantly along with increasing depths in all sediment profiles [42]. It was observed that the variability of the sulfur concentration at IN was greater than at OUT, in particular at RT1. The sulfur content at RT 1 IN showed the highest variability, which results mainly from the high content in the top layers. This can be linked to the decomposition of organic matter. The decrease in sulfur content with depth is typical, as the amount of organic matter decreases with depth, and with it the sulfur compounds [47]. Therefore, there is a reasonable suspicion that sulfur comes from sources other than the conversion of organic matter in the sediments.

### 3.4. Potassium Content

Potassium content in fertilizers is given as K_2_O. Therefore, our results for potassium content in bottom sediments are also presented in this form and are given in Table 3, showing the average value, standard deviation, and the min–max range. Sediments from RT 1 IN and RT 5 OUT had the highest K_2_O content of approx. 1.2%. A tendency to drop along with the vertical profile depth was also observed.

### 3.5. Calcium and Magnesium Content

Magnesium was measured as MgO. However, in Table 4, it is presented as elemental Mg. The bottom sediments from RT 8 OUT clearly had the highest Mg content along the whole vertical profile. In the case of RT3 and RT5, Mg was present in one layer of the sediment core only.

Calcium, similar to silicon, is one of the major constituents of bottom sediments in retention tanks. Calcium content was measured as CaO. However, in Table 5, it is presented as elemental Ca (after recalculation). The highest Ca content was measured at RT 3 OUT. In the case of RT1, RT3, and RT5, the IN values were higher than OUT along the whole sediment core. At RT8, this regularity was disturbed—only the top layer and the depth of 30 cm had a higher Ca content at IN.

## 4. Discussion

### 4.1. Analysis of the Relationships between Nitrogen and Organic Carbon Contents

The content of nitrogen in one-component and mutli-component fertilizers ranges between 14–30%. These numbers unfortunately confirm that the analyzed sediments are far too weak in nitrogen to be directly used as fertilizers. In addition to the content, the availability of nitrogen must also be taken into account. This can be analyzed by examining the carbon to nitrogen (C/N) ratio. It is important to keep the balance between the mineralization and immobilization processes. If this ratio is higher than 30, the microorganisms begin to take the available nitrogen from the soil, which then becomes insufficient for the plants. At a C/N ratio of 40, slow immobilization prevails, and when the ratio hits 50, immobilization is fast and complete. Otherwise, when the C/N ratio is lower than 20, the mineralization processes start to overwhelm the soil. As a result, the mineral forms of nitrogen (NH_4_, NO_3_) that can be readily uptaken by plants are formed. However, the excess of mineral nitrogen leaches from the soil or is transformed into gaseous products and lost [33]. Increasing the nitrogen content should not pose a major problem, as most of it comes from synthetic sources. For example, urea (CH_4_N_2_O) may be added to the sediment. By increasing the nitrogen content at the same time, it is necessary to increase the carbon content, which is currently too low. A very effective and proven solution, also economically justified, is the use of biochar [48]. The addition of pyrolysed straw, plant residues or others will significantly improve the fertilization capacity of the bottom sediments.

**Figure 8:**
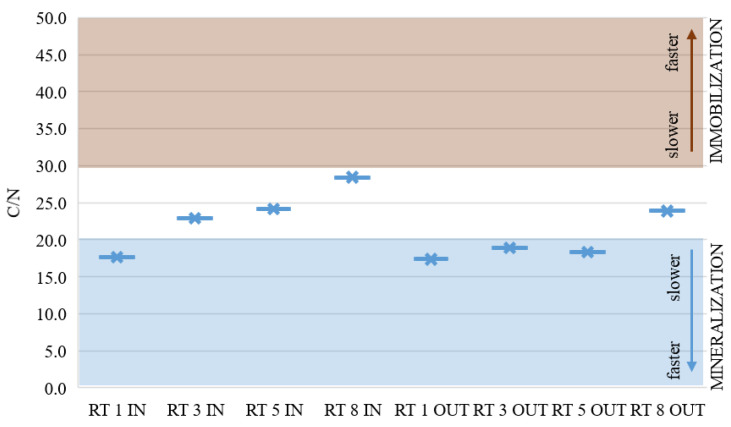
C/N average ratio in sediments from retention tanks in urban area [33].

Figure 8 presents the average of C/N analysis in the collected samples from eight points. In half of the samples taken, these ratios indicated a balance between mineralization and immobilization. In the remaining samples, these ratios were slightly lower and nitrogen would probably be available for the plants. These results show a good C/N ratio in the collected bottom sediments, but the N content is still too low.

### 4.2. The Impact of Sulfur on Nitrogen and Phosphorus Accessibility

The content of sulfur in the analyzed sediments varied from 0.01 to 2.46%. In RT1, RT2, and RT5, the higher sulfur contents were more present at the outflow than at the inflow. The major source of sulfur is gaseous SOx formed during the incineration of fossil fuels while generating energy and by vehicles. An additional source of S may be the decomposition of biomass. In the Oliwski Stream catchment, there are many old buildings from the beginning of the 20th century still using coal-fired boilers, which could be perceived as a potential source of SOx. As recently indicated by Nawrot et al. (2020) [9], coal combustion was the dominant source of Pb deposited in sediments of the Grunwaldzka retention tank (referred to as RT5 in this study) on Oliwski Stream, while in the case of retention tanks on another stream in Gdansk (Strzyza), a group of heavy metals containing Pb, Zn, Ni, Cr, and Fe originated partly from traffic and partly from coal combustion.

It was indicated that the optimal N/S ratio for plant growth is 15–16 for legumin plants and 11–12 for cereal plants, while for soil a ratio of 7 would be optimal [49]. Unfortunately, in the majority of the analyzed samples of bottom sediments, the nitrogen content was lower than sulfur. Therefore, the ratios were far lower than recommended, which means that the potential usage for soil fertilization would require an additional source of nitrogen. For instance, from urea. Despite this drawback, it is worth emphasizing that the sediments can be a valuable source of sulfur. The results show that the sulfur content is adequate even when the nitrogen content increases 60 times (growing cereals).

### 4.3. Analysis of Relationships between Phosphorus and Iron Content

The concentration of phosphorus compounds in water is strongly dependent on the presence of Fe ions. Table 6 presents the correlation coefficients between those two elements. The relevant correlations (*p* < 0.05) were confirmed for 6 out of 8 analyzed sediment cores. The correlation coefficients range from 0.71 to 0.92, which means that the correlation is high or very high.

The Fe/P ratio for each sediment core was also analyzed. According to many studies, the Fe/P ratio is an indicator of the P adsorption capability of the oxidized sediments, which is highly relevant, especially at the contact point between the water and sediments. The Fe/P ratio explains over 60% of P content in sediments [50].

The analysis of eight sediment cores showed that the Fe/P ratio in the top layers of sediments (0–10 cm) fluctuates from 2.2 to 7.3, with the lowest value at RT8 and the highest at RT1 (Table 7). For all RTs, the ratio values were higher at OUT than IN, which shows that phosphorus bioavailability was higher near the inflow. The average ratios for the whole sediment cores were above 2.0, which confirms that phosphorus could be leaching from sediments in the form of iron phosphates, which are available for plants. The high Fe/P ratio (on average above 2.0) also indicates that the sediments contained surplus iron that can contribute to the greater bioavailability of some additional phosphorus.

### 4.4. Analysis of Potassium Content in Sediments

The average content of potassium (in the form of K_2_O) in sediments was in the range of 0.30–0.81%. Animal manure is often used as a fertilizer for crops. For comparison, the potassium content similar to sediments can be found in duck and geese excrement. However, commercial fertilizers rich in potassium even contain 60% K_2_O, while P and K fertilizers contain 34% K_2_O, which means that the potassium content in the bottom sediments is too low.

### 4.5. Analysis of Magnesium and Calcium Content in Sediments

The calcium content in fertilizers is dependent on the agronomic category of soil and pH [51]. The maximum recommended calcium dose is 4 t CaO/ha. The appropriate preparation for sowing, including optimal stabilization of soil pH for each plant species, results in a crop increase of up to 60%. The analyzed bottom sediments have high contents of calcium. However, this would not be attractive enough since granules are the most recommended form of Ca supplementation, making it easier to control the distribution than the powdered form. The best type of calcium for agriculture is produced from limestone rocks with a CaO content up to 80%, while the maximum content in the sediments was 20%. Therefore, four times the amount of sediments would be needed to achieve the same Ca dose, which incurs additional costs of transportation, storage, and distribution, although the sediments are available for free. Often, calcium-magnesium fertilizers are used, which contain dolomite calcium with the additive of magnesium. They are characterized by lower reactivity. Another possibility is to use waste material containing calcium and magnesium and this could be a chance to use bottom sediments. However, the magnesium content (in the form of MgO) in commercial fertilizers is approx. 15%, while the analyzed sediments only contain 2.33% MgO, which is six times lower.

### 4.6. Comparison of the Composition of the Bottom Sediments of the Retention Reservoirs to the Bottom Sediments of Fish Farming Ponds

In order to compare the obtained results with previous similar studies, the quality of the bottom sediments of municipal retention reservoirs [5,52] was compared with the analysis of sediment composition from fish ponds [52]. The average nitrogen content in the fish pond sediments was 0.2% and ranged from 0.13–2.86% depending on the type of aquaculture with a median closer to 0.3%. This result was about four times higher than in the sediments from the reservoirs from the Oliwski Stream. Hauqe et al. (2016) [5] reported that the content of organic carbon in the sediments of pangasius fish ponds was 3.15%, which is about 20% more than in urban reservoirs. In fish ponds, the C/N ratio varied between 9.1–18.85, which is unfavorable for fertilization due to nitrogen mineralization. In this case, a better C/N ratio was obtained for bottom sediments in this study. The phosphorus content in fish pond sediments was 0.13% on average, which is almost equal to the value obtained for urban retention tanks. The potassium content in fish ponds was only 0.10%, which is about five times lower than in urban sediments [53]. Different results occurred for the measurements of P and K in ponds with pangasius breeding, where the contents were 11.6 and 10.7%, respectively [5]. The sulfur content in the studies by Haque et al. was also high (8.6%), while the maximum result for urban tanks was only 2.5%. In the study by Muendo et al. [52], the composition of the sediments was even worse than in the studies by Kouba et al. [53] and Eymontt et al. [6] with the exception of high organic carbon content. To summarize, the bottom sediments of retention reservoirs are not worse than the aquaculture sediments in terms of phosphorus or C/N ratio. However, the variability in the composition of fish pond sediments is significant, which makes the comparisons a rather difficult task.

### 4.7. The Legal Possibilities of Using Bottom Sediments for Soil Fertilization

Bottom sediments can collect many elements, including potentially toxic ones such as heavy metals (HM). Based on the heavy metal concentrations in bottom sediments from some retention tanks on Oliwski Stream, which are reported by Nawrot et al. (2021) [54], the concentrations of Cd, Cu, Ni, Pb, and Zn were lower than recommended by the Council Directive on the protection of the environment, and in particular for soil, when sewage sludge is used in agriculture [30] (Table 8). There are some additional guidelines worth taking into account, as followed by Amlinger, Pollak, and Favoino (2004) [55]. They compared HM concentration in lake sediment to the limit values used for class A compost [55]. The results of the analysis again indicated that the concentration of heavy metals does not limit the use of sludge in agriculture. The analysis carried out also included tests of mercury content (required by the same Directive) and this element was not detected in any of the samples taken. The analysis carried out between the HM content in the samples taken and the limits of the Directive cannot be dismissed as a potential source of plant growth enhancer.

### 4.8. Proposition of Selection Criteria for Bottom Sediments to Be Applied for Soil Fertilization

Results from the analysis of elemental contents and ratios of the sediment cores from urban retention tanks were compared using a multi-criterion analysis to identify those which are the most promising in terms of their fertilization potential. Three criteria were defined (Formulas (1)–(3) are shown below): Criterion (1) includes the content of major nutrients N, P and K, and organic carbon, (2) refers to the content of additional elements: Fe, S, Ca, and Mg, while (3) includes the Fe/P and C/N ratios. Each criterion received its own weighing factor, listed in Table 9. The weighing factor of the criterion (1) is higher than (2) and (3) since it refers to the contents of major nutrients and organic carbon, which are indispensable for growth of plants. Therefore, their concentrations in the fertilizing material should be higher than the other elements. For criteria (1) and (2), the maximum scores were given to the sediment core with the highest elemental contents. Each element (N, P, K, and Corg) inside the criterion (1) were ranked equally (0.25) and the maximum value was 8 (8 sampling points). For criterion (2), the contents of Fe and S received a weight of 0.35 each, while the weight for Ca and Mg was 0.15. For criterion (3), both the Fe/P and C/N ratios received an equal weight of 0.5. Criterion (3) takes into account the ratios that are most favorable in terms of using sludge as a fertilizing material, and thus the availability of phosphorus and nitrogen for plants. Those with the best availability received the maximum number of points. The calculation scheme is presented by Formulas (1)–(4). All of the analyses were performed using the average content in the core.


(1) Crit. 1=0.25 · P+0.25 · N+0.25 · Corg+0.25 · K 

(2) Crit. 2=0.35 · Fe+0.35 · S+0.15 · Mg+0.15 · Ca 

(3) Crit. 3=0.5 · Fe/P+0.5 · C/N 

(4) SUM=0.4 · Crit. 1+0.3 · Crit. 2+0.3 · Crit. 3

The results of the multi-criteria analysis are presented in Table 10. Sediments from RT 5 OUT received the highest score, corresponding to the best fertilization potential, followed by the sediments from RT 8 OUT. The sediments from RT 3 IN received the lowest score, substantially lower than the other analyzed cores. Another approach involved combining (summing) the results from IN and OUT for each retention tank (Table 11), which seems to be reasonable from a practical point of view. When dredging reservoirs, it can be difficult to separate the sediments collected from the inflow and outflow. It is also worth checking whether mixing the sludge will significantly change the order given in the previous table. Using this approach, RT 5 received the highest score, followed by RT8, RT3, and RT1.

## 5. Conclusions

In this study, an attempt was made to answer if the bottom sediments dredged from urban retention tanks, currently perceived as waste to be managed, can be a potential fertilizing material supporting plant growth. Without a doubt, these aspects should be examined with respect to the composition of sediments from each site. Our analyses showed that the elemental composition of sediments varied substantially among the analyzed retention tanks and even for IN and OUT sampling sites at each tank. Therefore, a simple tool to compare the fertilizing potential of sediments is proposed. Of all the analyzed sediments, the sediments from RT5 scored the highest, while the sediments from RT1 were the poorest, probably due to the type of management of the catchment area. However, this is a topic for further analysis. The results from our study show that the bottom sediments have low contents of nitrogen and organic carbon. Therefore, it is recommended to enrich the sediments with sources of nitrogen and organic carbon. An example is urea or biochar, which is a low-cost ingredient. The great advantage of bottom sediments is a high content of iron, maximally even 3.3%, which translates into the high bioavailability of phosphorus for plants. The sediments are also rich in sulfur (maximum 2.5%), which is relevant since worldwide sulfur resources are scarce. Considering the contents of calcium and magnesium (maximum od Ca was 14.47%, and of Mg 2.01%), the sediments contain four and six times lower amounts of these elements, respectively, than commercial fertilizers. This again implies the need to supplement or apply respectively higher doses of sediments.

To summarize, bottom sediments should not be used directly as a fertilizer. They should be enriched with at least nitrogen and organic carbon. Bottom sediments are not a well-balanced fertilizer mixture, but they are a valuable source of some elements (iron and sulfur). The biggest disadvantage of bottom sediments in the context of their use in agriculture is the low concentration of phosphorus, which is a non-renewable resource. Finally, bottom sediments can be potentially used in agriculture after enrichment. Future research should focus on selecting a low-cost material to supplement the sediments and on performing cultivation experiments to select the plant type, dose, and method of applying bottom sediments to ensure the highest possible recovery of elements necessary for plant growth.

## Figures and Tables

**Table 1:** Characteristics of the selected retention tanks.

No.	Retention Tank Name	Distance from the Mouth [km + m]	Tank Area [ha]	Reservoir Tank Capacity [m^3^]
1	Reservoir No. 1, Jelitkowska Street	0 + 327	1.01	5050
3	Reservoir No. 3, Chłopska Street	1 + 411	1.20	6000
5	Reservoir No. 5, Grunwaldzka Street	2 + 819	1.69	8450
8	Reservoir No. 8, Spacerowa Street	4 + 010	1.08	5040
SUM			13.51	70,797

**Table 2:** Geochemical background of Ca, Fe, Mg, P, and S in the soils of the studied area [44].

Elements	Ca	Fe	Mg	S	P
Geochemical background [%]	0.46	0.65	0.09	0.022	0.060
Scattering of the results [%]	0.00–14.47	0.16–3.30	0.00–1.40	0.00–2.46	0.00–0.36

**Table 3:** Potassium content (K_2_O) in samples of bottom sediments from RT.

Sampling Point	Min	Max	Average	Standard Deviation
RT 1 IN	0.38	1.21	0.79	0.29
RT 1 OUT	0.18	0.98	0.66	0.25
RT 3 IN	0.00	1.05	0.78	0.28
RT 3 OUT	0.09	0.81	0.53	0.22
RT 5 IN	0.25	0.69	0.52	0.15
RT 5 OUT	0.35	1.20	0.81	0.25
RT 8 IN	0.00	0.88	0.52	0.20
RT 8 OUT	0.06	0.43	0.30	0.11

**Table 4:** Magnesium content in bottom sediments of municipal retention tanks.

Sampling Point	Min	Max	Average	Standard Deviation
RT 1 IN	0.00	1.10	0.48	0.42
RT 1 OUT	0.00	1.10	0.61	0.34
RT 3 IN	0.00	0.95	0.61	0.31
RT 3 OUT	0.00	1.21	0.09	0.31
RT 5 IN	0.00	1.04	0.67	0.35
RT 5 OUT	0.00	0.84	0.09	0.27
RT 8 IN	0.00	0.90	0.65	0.31
RT 8 OUT	0.75	1.40	1.00	0.16

**Table 5:** Calcium content in bottom sediments of municipal retention tanks.

Sampling Point	Min	Max	Average	Standard Deviation
RT 1 IN	0.64	6.97	2.62	2.31
RT 1 OUT	2.31	11.00	6.49	2.57
RT 3 IN	0.00	6.17	3.61	1.61
RT 3 OUT	4.66	14.47	11.03	2.88
RT 5 IN	1.15	4.52	2.75	1.01
RT 5 OUT	4.96	11.99	8.84	2.16
RT 8 IN	0.00	7.03	3.15	1.57
RT 8 OUT	2.05	6.04	3.58	1.45

**Table 6:** Correlations between P and Fe at eight sampling points.

Ratio	RT 1 IN	RT 1 OUT	RT 3 IN	RT 3 OUT	RT 5 IN	RT 5 OUT	RT 8 IN	RT 8 OUT
Fe/P	0.79	0.80	0.92	0.23	0.56	0.71	0.92	0.90

**Table 7:** Characteristic values of Fe/P ratio coefficient in sediments from the analyzed RTs.

Sampling Point	RT 1 IN	RT 1 OUT	RT 3 IN	RT 3 OUT	RT 5 IN	RT 5 OUT	RT 8 IN	RT 8 OUT
MIN	4.0	6.6	0.0	8.8	5.2	9.8	0.0	6.8
MAX	23.1	13.7	10.8	14.6	15.6	18.8	9.6	12.1
Median	9.5	8.9	7.2	10.3	9.1	13.1	6.7	8.6
Average	11.0	9.7	7.0	10.8	9.2	13.1	6.6	8.9
Standard deviation	5.2	2.3	2.5	1.9	2.9	2.8	2.4	1.7

**Table 8:** Comparison of HM content in bottom sediments from Oliwski Steam tanks [54,56] with the limits given in Council Directive [30] and compost “class A”, which is suitable for agriculture in Europe [55].

Heavy Metals	Limit Values for Heavy-Metal Concentrations in Sludge for Use in Agriculture	Compost A	Content in Bottom Sediments in RT in Oliwski Stream
	[mg/kg Dry Matter]		
Cd	20–40	1	0.091–0.469
Cu	1000–1750	150	37.8–64.9
Ni	300–400	60	3.80–10.30
Pb	750–1200	120	22.7–81.9
Zn	2500–4000	500	45.0–244
Hg	16–25	0.7	not detected
Cr	-	70	not detected
As	-	23	not detected

**Table 9:** Criteria of quality evaluation of sediment cores.

Criterion	1	2	3
Criterion description	Contents of P, N, Corg, K	Contents of Fe, S, Mg, Ca	Ratio Fe/P, C/N
Weight factor	0.4	0.3	0.3

**Table 10:** Results of the multi-criteria analysis of bottom sediments.

No.	Sampling Point	SUM of Crit. 1–3
1	RT 5 OUT	4.74
2	RT 8 OUT	4.05
3	RT 8 IN	3.37
4	RT 5 IN	3.26
5	RT 1 IN	3.22
6	RT 1 OUT	2.99
7	RT 3 OUT	2.98
8	RT 3 IN	2.11

**Table 11:** Results of the multi-criteria analysis for the whole retention tank (sum of In and OUT).

No.	Retention Tank	SUM of IN and OUT
1	RT 5	8.0
2	RT 8	7.4
3	RT 1	6.2
4	RT 3	5.1

## Data Availability

The data presented in this study are available on request from the corresponding author.

## Abbreviations

Ntot	Total nitrogen
Corg	Organic carbon
RT	Retention tank
IN	Inflow (to retention tank)
OUT	Outflow (from retention tank)
HM	Heavy metal
Crit.	Criterion
